# An adjustable wave model for attention restoration

**DOI:** 10.3389/fcogn.2026.1842241

**Published:** 2026-07-21

**Authors:** Richard J. Jagacinski

**Affiliations:** Department of Psychology, Ohio State University, Columbus, OH, United States

**Keywords:** attention restoration theory, distributed attention, music, religious practice, sampling, traveling wave, vigilance decrement, language disorders

## Abstract

Attention Restoration Theory has provided a framework for investigating how experiencing natural scenes can be especially helpful in overcoming attentional fatigue. If the structure of attention were better understood, it might facilitate experimental investigations of this theory and possibly expand its domain. The present paper reviews experiments that demonstrate that sustained focal attention is characterized by 3–8 Hz. oscillations that are interpreted as temporally sampling the environment. A traveling wave model is proposed as a generalization of this finding for distributed attention in which there is sampling of the environment over both time and space. Temporal and spatial sampling frequencies are assumed to be adjustable together from low to high. This model provides a frequency based interpretation of Attention Restoration Theory. Low frequency attention waves can occur in expansive natural environments with slow temporal rhythms and fractal structure and are restorative. In contrast, high frequency attention waves can occur in modern technological environments with high event rates and are fatiguing. This model of distributed attention will be helpful in researching attention restoration in relation to the spatio-temporal frequency content of natural and constructed environments. Attention restoration is discussed in the context of monitoring and vehicular control tasks, attentional aspects of religious practice, and music.

## 1 Introduction

People's ability to perform cognitively demanding information processing tasks deteriorates over time (e.g., Warm et al., 2008). However, controlled experiments indicate that performance can be restored to a greater degree after a walk through a natural scene such as an arboretum than after a walk through a cityscape (Berman et al., 2008; Kaplan and Berman, 2010). Findings such as these (Ohly et al., 2016; Stevenson et al., 2018; Bell et al., 2025) have provided empirical support for Attention Restoration Theory (Kaplan and Kaplan, 1989).

There are many properties of natural scenes that make them different from constructed environments. Nature and images of natural scenes can generate feelings of mystery and awe (e.g., Kaplan and Kaplan, 1989; Marois et al., 2021). It has been theorized that the attention underlying such reactions is different from much of daily attention. Kaplan and Kaplan (1989) have theorized that nature is intrinsically fascinating and engenders a “soft” (non-intense) form of involuntary, effortless, bottom-up attention that is different from intense involuntary attention and different from voluntarily directed, top-down attention, which is effortful to various degrees. “Bottom-up” refers to the environmental shaping of attention and is contrasted with “top-down,” which refers to cognitively directed attention. Attention Restoration Theory claims that by engaging involuntary, effortless, bottom-up attention, natural scenes greatly relieve voluntarily directed top-down attention and thereby allow voluntarily directed, top-down attention to be restored from fatigue (Kaplan and Kaplan, 1989).

According to Kaplan and Kaplan (1989, pp. 183–186), four aspects of natural scenes that facilitate attention restoration are: (1) Being Away from the attentional demands of daily tasks and from distractions; (2) Large Extent or scope that reveals meaningful connections or interrelations among the elements of a scene, for example, “a whole other world”; (3) fascination that arises from inherently interesting and/or mysterious patterns; and (4) compatibility between the pre-existing goals or motivations of the person and the actions afforded by the environment. The present discussion of the structure of attention will emphasize Being Away from task-imposed attention to detail and from distractions, Extent as it relates to expansive natural scenes and tall buildings, and Fascination for its implied low level of intensity and effort.

One criticism of the original formulation of Attention Restoration Theory has come from Marois et al. (2021), who measured eye movements and pupil dilation in viewing natural scenes that varied in mystery. They concluded that attentional restoration is due to bottom-up attention and directed, top-down attention actively engaging together to process natural scenes. This research aligns with Kaplan's (2001) previous suggestion that goal directed attention in processing a natural environment could enhance its restorative effectiveness. This statement is a departure from the original formulation of Attention Restoration Theory. Another criticism of Attention Restoration Theory is that attention related concepts such as fascination are qualitative and/or difficult to measure (e.g., Joye and Dewitte, 2018; Celikors and Wells, 2022).

Attention Restoration Theory could benefit from a more quantitative model of the processes underlying recovery from cognitive fatigue. A wave model of distributed attention is therefore proposed as a more quantitative construct in which slow attentional frequencies are the key restorative element. Both spatial and temporal frequencies in the wave model are assumed to be influenced by various bottom-up environmental stimuli as well as top-down cognition. These two types of frequency are proportional to each other in a traveling wave, so that slowing one will slow the other. This theoretical perspective will be shown to be supported by experiments on dynamic aspects of perception and attention, by behavioral measures of slowing, by subjective reports of how people “slow down” in natural environments, by reactions to tall architecture, by rhythmic meditative practices, and by music that people find to be restorative. Remediation of cognitive fatigue from extended vigilance tasks and from aspects of modern technology will also be discussed. The present article will first describe a traveling wave model of distributed attention in more detail and then review various behavioral data that make this perspective a likely candidate for guiding future research on the restoration of attention.

## 2 A wave model of distributed attention: large distances and slowing down go together

**Figure 1:**
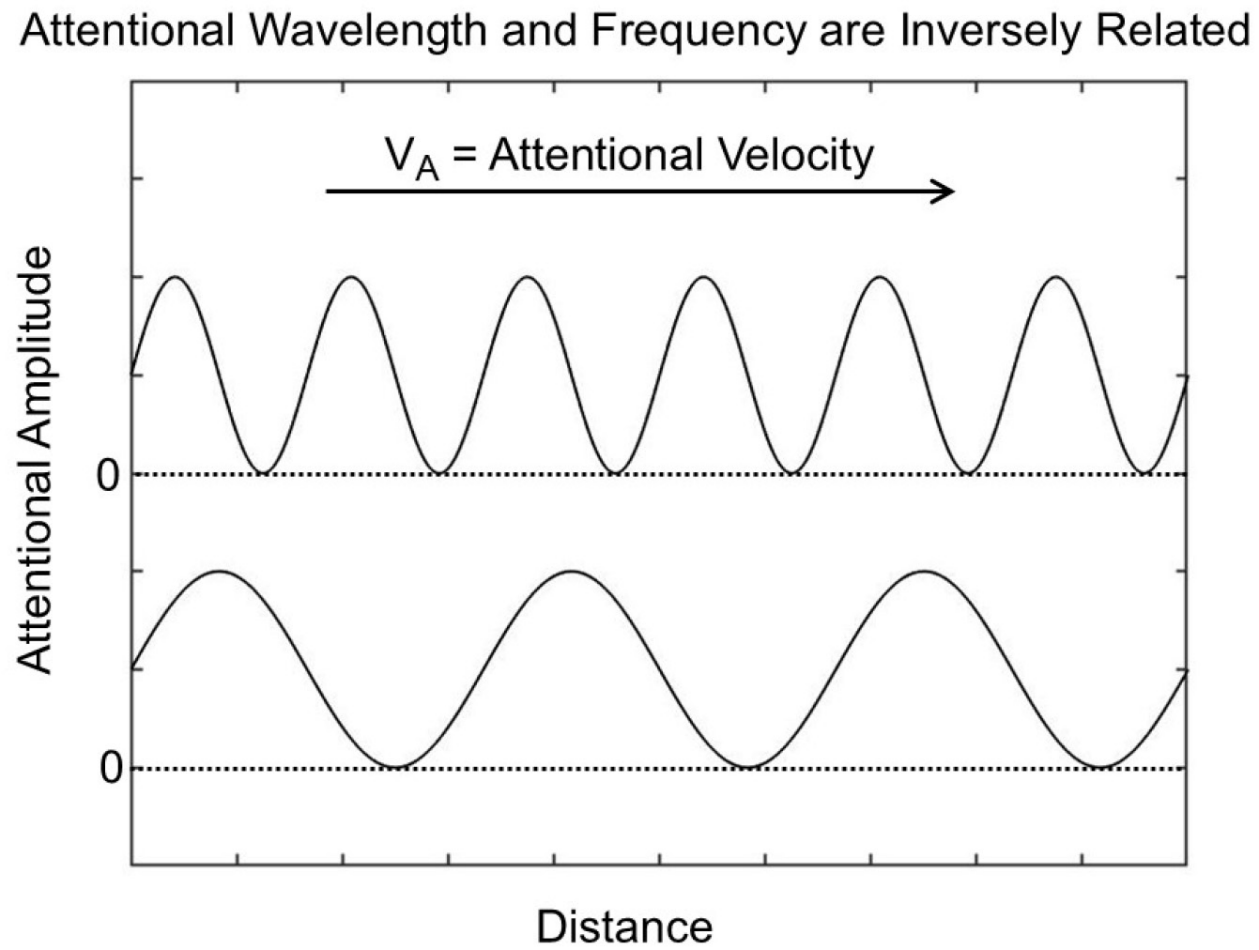
The wavelength of an attention wave is the (horizontal) distance between successive high peaks. Distance refers to the perceptual representation of spatial extent in a scene. **Top**: a short wavelength, high spatial frequency attention wave. **Bottom**: a long wavelength, low spatial frequency attention wave. As the spatial wavelength of an attention wave increases as might occur in expansive natural environments, its spatial frequency and proportionally related temporal frequency decrease. Both the upper and lower waves are traveling at the same constant velocity, V_A_. The figure represents an edge view of a two-dimensional wave that extends perpendicular to the plane of the figure. The vertical separation of the two waves is for illustrative clarity only.

A wave metaphor for distributed attention postulates a constant velocity and adjustable wavelength and cycle time (Jagacinski et al., 2024). In a wave that is traveling at a constant velocity, a longer wavelength means that the distance and time between successive high peaks will be longer (Figure 1). In other words, as attentional wavelength increases as might occur in expansive natural settings, both the spatial and temporal frequencies of attention will decrease. It is this co-occurring decrease in attentional frequencies that people may be referring to when they report “slowing down” in expansive natural environments (e.g., Holden, 2025). Experimental support for the various features of this model will now be summarized.

### 2.1 Multiple peaks of attention

One qualitative feature of this model is that distributed attention has multiple traveling peaks spread out in space, which allows high levels of attention to occur at multiple separated locations simultaneously. The low troughs between the high peaks in Figure 1 represent little or no attention. Distributed attention is qualitatively different from highly focused attention, which has been described as a single localized spotlight that moves from place to place sequentially (e.g., Eriksen and St. James, 1986). Evidence for this distinction comes from visual search tasks. If the time to find a target stimulus increases with the number of items to be searched, that temporal pattern suggests the search is progressing as if a spotlight were moving sequentially from item to item until it comes to the target. This increase in search time with increasing number of items is found when the target is a conjunction of two or more stimulus features such as a green letter T (Treisman and Gelade, 1980). However, if the time to find a target stimulus is relatively constant with increases in the number of items to be searched, that temporal pattern suggests that the entire array of stimuli is being processed simultaneously (Treisman and Gelade, 1980; Treisman, 2006). This pattern is found when the target is an individual feature such as a particular color or shape. Subjectively the target seems to “pop out” from the surrounding distractors. A planar two-dimensional wave of attention that is rapidly traveling over the entire array of stimuli could approximate this simultaneous processing and would be an example of distributed attention. Estimating the mean size of a set of objects provides another example of distributed attention (Treisman, 2006; Baek and Chong, 2020). The present wave theory assumes that attention restoration is primarily associated with low frequency distributed attention as might be used to perceive the gist of a scene (Treisman, 2006), and not with highly focused attention as would be used to identify a detailed object.

### 2.2 Attentional velocity determines the proportionality of spatial and temporal frequencies

A second feature of the wave model is that its constant velocity implies a proportional relation between wavelength and cycle duration across different waves. A simple equation for explaining the wave structure is


$$\begin{array}{l} Attentional\;velocity\;=\;Wavelength/Cycle\;duration \end{array}$$ (1)

The distance covered in one cycle (Wavelength) divided by the amount of time it takes to do so (Cycle time) is equal to the velocity of a wave.

The Temporal frequency of a wave equals 1/Cycle duration. Equation 1 can therefore be rewritten as


$$\begin{array}{l} Attentional\;velocity\;=\;Wavelength\;\times \;Temporal\;frequency \end{array}$$ (2)

Attentional velocity is assumed to be constant. Therefore, Wavelength and Temporal frequency must vary inversely.

The Spatial frequency of a traveling wave equals 1/Wavelength, and therefore 1/Spatial frequency equals Wavelength. Equation 2 can therefore be rewritten as


$$\begin{array}{l} Attentional\;velocity\;=\;Temporal\;frequency/Spatial\;frequency \end{array}$$ (3)

The ratio of Temporal frequency to Spatial frequency of a traveling wave is equal to its Attentional velocity. Therefore, in Figure 1 the top wave has both a higher Spatial frequency and a higher Temporal frequency than the wave at the bottom of Figure 1. Equations 1–3 are simply different ways of describing a space-time property of waves with constant velocity. Although time and space are often conceptualized as independent entities from the perspective of Newtonian physics, the wave model of distributed attention represents a type of psychological space-time coupling (see also Jones, 1976). As Spatial frequency decreases, so does Temporal frequency.

Evidence for a constant attentional wave velocity comes from the boundary between perceiving a sequence of brief lights as a single movement trajectory at slower event rates (focused attention) and perceiving that same sequence as multiple simultaneous trajectories at faster event rates (distributed attention). The latter effect is known as perceptual streaming (Bregman, 1990). Extrapolating from research on the perception of apparent motion, Klapp (in Klapp et al., 2019, p. 40) suggested that the transition to multiple simultaneous trajectories as a light sequence is sped up indicates a velocity limit on the movement of attention beyond which the single spotlight of focused attention can no longer keep up with the sequence of events. For example, if a sequence of briefly displayed lights forms a repeating rectangular pattern, at slower speeds (longer light durations) it is perceived as the apparent motion of a single light moving from one corner of the rectangle to another. However, at higher speeds (shorter light durations) the same display is perceived as two simultaneous back and forth horizontal motions at the top and bottom of the rectangle (Jagacinski et al., 2024). In other words, there is a transition from a single trajectory at slower speeds to two simultaneous trajectories at higher speeds, a transition from a moving spotlight of focal attention to attention that is simultaneously distributed to two separated locations. The duration of the briefly displayed lights at this transition increases when the height of the rectangle increases, and the plot of rectangle height vs. light duration at transition is highly linear. The ratio of the increase in rectangle height to the increase in light duration can therefore be interpreted as a velocity limitation on the movement of focal attention and also the constant velocity of distributed attention (Jagacinski et al., 2024). A similar phenomenon occurs in the auditory domain at the boundary between hearing a sequence of tones as a single melodic sequence or hearing multiple simultaneous melodies, which has also been interpreted as a velocity boundary (e.g., van Noorden, 1975; Jones and Yee, 1993).

### 2.3 Attentional oscillations indicate environmental sampling

A third qualitative property of the wave model is that at any fixed position in space, attention will oscillate temporally as the wave passes by. This property agrees with experiments in which an observer had to detect a faint signal at a primed location when there was temporal uncertainty as to when the signal would occur. The hit rate in detecting the signal was found to oscillate in a range from about 3 to 8 Hz (Helfrich et al., 2018; VanRullen, 2018; Fiebelkorn and Kastner, 2019). These studies involved sustained focal attention and inferred that it was oscillatory. Similarly, Jagacinski et al. (2024) inferred temporal attentional frequencies from 3 to 6 Hz. at the transition boundary from focal to distributed attention when a rectangular sequence of brief lights was sped up as described above. 3 Hz corresponds to 333 ms between successive attentional peaks, and 8 Hz. corresponds to 125 ms between successive attentional peaks. Although such rapid oscillations in attention are not subjectively noticed, the proportionally large change from high to low frequencies may result in noticeable behavioral slowing that is restorative.

Although the present paper will concentrate on behavioral measures of performance, physiological correlates of behavior can provide converging measures and complementary insight into the underlying mechanisms of behavior. For example, the phase of localized intracranial EEG theta waves in the 3–8 Hz. range has been found to be highly correlated with oscillations in the hit rate in detecting faint visual signals (Helfrich et al., 2018; VanRullen, 2018; Fiebelkorn and Kastner, 2019). Temporal oscillations in hit rate are thus correlated with EEG oscillations, which can therefore be interpreted as variations in attention. These signal detection tasks involved focal attention to small regions of space, and have been interpreted as temporal sampling of the environment (Busch and VanRullen, 2010). The newly proposed wave model (Jagacinski et al., 2024) can be considered a generalization of this interpretation to distributed attention in which there is rhythmic sampling of the environment in both time and space. The ratio of the temporal and spatial sampling frequencies is determined by the velocity of the traveling wave (Equation 3). Physiological evidence for electrical traveling waves has been found in many parts of the brains of different species (e.g., Muller et al., 2018). The present wave model predicts that at least some of these traveling waves will correlate with behavioral measures of distributed attention in a manner which parallels the results for focal attention. Also predicted is that physiological measures of attentional frequency will be shown to be adjustable. However, it should be noted that physiological measurement of localized EEG's (e.g., Helfrich et al., 2018) and localized traveling waves (Benucci et al., 2007; Muller et al., 2018) typically involves invasive, under-the-skull, intracranial techniques in order to get high resolution measurement. The invasive nature of this research makes it difficult to conduct. Another aspect of the present wave model is that it involves temporal sampling intervals less than 1 s, which corresponds to a low level in Rosenberg's (2026) hierarchical temporal model of attention. As reviewed by Rosenberg, there are many other physiological correlates of attention over longer temporal intervals including various network structures, and these are beyond the scope of the present paper. The higher levels in Rosenberg's hierarchy involve slower processes that may be involved in complex feelings such as awe and mystery, which are another aspect of Attention Restoration Theory (e.g., Marois et al., 2021).

An alternative approach to invasive physiological measurement that is more amenable to behavioral testing is to postulate a quantitative wave model and how it is coupled to environmental and cognitive influences, and see if the pattern of behavioral change to these influences is as predicted (e.g., Cardoso et al., 2025). In particular, can the wave model provide a sufficiently abstract description of the restorative experience of a walk in the woods that it can be generalized to other physically different settings and thereby predict which other different settings and/or activities will be highly restorative? Can the wave model critique present techniques for measuring attentional restoration? Can the abstraction of the wave model encourage dialogue across different subdisciplines of behavioral research that may enrich each other? These are tests of this model at a behavioral level.

### 2.4 Environmental and cognitive influences on attentional frequency

A fourth feature of the wave model is that the spatial and temporal sampling frequencies, which correspond to the occurrence of attentional peaks in the traveling waves, are assumed to be influenced by both environmental stimuli and by cognitive control. The latter influence implies that people are sensitive to the spatial and/or temporal structure of the environment and can adjust their attentional frequencies accordingly. Support for this assumption comes from Senders (1964; summarized in Sheridan and Ferrell, 1974). He studied people monitoring multiple visual displays of time varying signals to detect whenever a pointer exceeded some specified level on any of four dials. People adaptively adjusted their eye movements to increase their sampling rates of the signals with higher bandwidths. The Nyquist-Shannon sampling theorem indicates that a continuous signal can be reconstructed from discrete samples collected at a rate that is at least twice the bandwidth of the continuous signal (e.g., Wikipedia, 2026). Senders (1964) found that people's eye movement sampling rates were highly correlated with this criterion. People were thus intuitively sensitive to the relation between signal bandwidth and sampling frequency, i.e., more quickly varying signals need to be sampled more frequently.

An example of attentional adjustment to temporal environmental frequencies comes from Jones et al. (2002). They found that presenting a uniformly paced sequence of tones improved people's ability to make judgements of pitch differences when the timing of the test tone was consistent with the preceding sequence. Their interpretation was that attention was rhythmically entrained to the regularly paced preceding sequence. Large and Jones (1999) provided a quantitative theory of entrainment for interpreting these results by positing internal adjustable attentional oscillators. The oscillators produce appropriately timed pulses of attentional energy by adjusting their temporal frequency, phase, and pulse width to achieve synchrony with external rhythmic tones. These studies show that people are sensitive to temporal environmental frequencies and can adjust their attention in appropriate ways to facilitate performance.

An example of attentional adjustment to spatial environmental frequencies is provided by Abrams and Weidler's (2014) research on attention to space near the hands, i.e., perihand space. People had to judge whether visual gratings with a particular spatial frequency were slightly titled to the left or right. When the gratings were positioned close to the observer's hands, people were more sensitive to tilt when the grating frequency was lower. When the gratings were not close to the observer's hands, people were more sensitive to tilt when the grating frequency was higher. These results can be interpreted as attentional emphasis of lower spatial frequencies in regions of space where the person could make hand movements. The underlying physiological mechanism is thought to be adaptive adjustment of magnocellular vs. parvocellular visual processing (Abrams and Weidler, 2014).

Sensitivities such as those demonstrated in the above three experiments are part of the wave model of distributed attention, which postulates that attentional frequencies can be adaptively adjusted. For example, attentional frequencies might be adjusted by attending to either spatial or temporal aspects of the environment. Looking at expansive natural scenes may increase attentional wavelength and thereby decrease both spatial and temporal attentional frequencies. For example, in a book that extolls the benefits of slowing down, Holden (2025, p. 60) describes how he enjoys watching the sun set from an ocean beach each evening. The distant view of the sunset could induce a long attentional wavelength. Additionally, listening to the rhythm of the ocean waves could induce a long attentional cycle duration that would also contribute to low spatial and temporal attentional frequencies.

Other meditative practices may also decrease attentional frequencies in a more top-down fashion. Although the original formulation of Attention Restoration Theory emphasized bottom-up influences on attention, in later years Kaplan (2001, pp. 499–500) considered experiencing nature and meditation as complementary ways to restore voluntarily directed attention. They could be effective individually, or they could combine to increase attention restoration even more effectively together. Similarly, attention waves are a theoretical construct that treats experiencing nature and meditation as complementary ways to achieve low frequency attention. Long attentional cycle durations might be implemented by performing and attending to slow breathing as in mindfulness practice, slow bodily movements as in Qi Gong, or by other meditative practices (e.g., Holden, 2025; Zaleski, 1995). These behaviors are generally considered restorative. It is the slowing of attentional frequencies in these various ways that may be a key element in attention restoration.

## 3 On being expansive, slow, fractal, and free of distractions

The high peaks in the wave structure of distributed attention in Figure 1 can be considered as successive samplings from various perceptual systems over time and space. More frequent sampling in time and space can be used to make finer distinctions among environmental stimuli and may be more cognitively fatiguing. In contrast, lower frequency attention waves involve less frequent samplings and may therefore be restorative.

The issue of why low frequency attention may be restorative suggests an analogy with the physics of light waves. Visible light waves all have the same constant velocity but vary in spatial and temporal frequency. Low frequency visible light waves are perceived as having reddish color. High frequency visible light waves are perceived as having violet color. The energy of a light wave is proportional to its frequency. Therefore, low frequency red light waves have less energy than high frequency violet light waves (e.g., Lincoln, 2017). Similarly, low frequency attention waves may require less energy or effort than high frequency attention waves. Low frequency attentional sampling may also unburden higher level attentional processes due to the lower effective bandwidth of environmental information.

Three properties that may contribute to natural forest scenes being unusually restorative are (1) their large spatial extent and slow temporal rhythms which facilitate slow attentional frequencies, (2) fractal structure which makes low frequency approximations to the environment informative, and (3) a lack of sudden distractions which would encourage high frequency attention to particular regions of space. In terms of Attention Restoration Theory, (1) and (2) can be considered aspects of Extent, which entails both scope and connectedness, and (3) can be interpreted as Being Away from the distractions of daily life.

Regarding property (2), one can ask whether natural environments have structural features that facilitate the lowering of attentional frequency? One aspect of natural scenes is that they typically exhibit strong structural similarities across different levels of magnification, which is referred to as “fractal” (Mandelbrot, 1983) and can be considered a form of “connectedness” in terms of Attention Restoration Theory. Others have suggested that this structural feature may make an important contribution to attention restoration (e.g., Purcell et al., 2001; Joye, 2007; Schertz and Berman, 2019). Similarity at different levels of detail can be quantified by analyzing scenes with Fourier and/or wavelet analysis (e.g., Field, 1993), which decompose scenes into spatial and/or temporal frequencies with differing amplitudes. Slower attention wave frequencies will emphasize coarser, lower bandwidth approximations to the environment (e.g., Yantis and Abrams, 2017, p. 275). However, in natural environments with strong self-similarity in different frequency bands, for example, Field (1993, p. 172), one can restrict the effective environmental bandwidth with slow sampling and still obtain important structural information. Therefore, such environments afford lowering the sampling frequency better than environments that have accentuated high frequency bands and therefore place greater demands on cognitive processes. The wave model of attention thus provides a process oriented explanation of why fractal structure may facilitate attention restoration.

Regarding property (3), Kaplan and Kaplan (1989) described restorative attention as non-intense, involuntary, effortless, bottom-up attention that greatly relieves voluntarily directed, top-down attention. Joye and Dewitte (2018) have questioned how Attention Restoration Theory can specify whether nature's fascination is soft (non-intense) enough so as to permit restoration, or too intense and therefore not restorative. The wave theory of attention can conceptually address variations in experiential intensity by considering the spatio-temporal structure of the environment. Intense interactions with nature such as white water rafting, dodging a tornado, or being outdoors in a thunderstorm are likely to attract high frequency attention to rapid, localized, high energy events and therefore not be restorative. Fourier and/or wavelet analysis of such environments will reveal heightened high frequency temporal and spatial components corresponding to sudden changes over space and time (Fourier analysis is analogous to performing multiple regression with sinewaves to see which frequencies are needed to approximate the environment, e.g., Jagacinski and Flach, 2009). Similarly, an urban environment with sudden, localized distracting events requiring immediate high frequency attention will not be very restorative (Berman et al., 2008; Kaplan and Berman, 2010). Characterizing environments in terms of their spatial and temporal frequencies and energy levels can provide a step toward answering Joye and Dewitte's challenge.

Attributing the restorative power of nature to its promotion of slow attentional frequencies raises the possibility of opening dialogues between researchers of attention restoration and researchers in other disparate fields of research, for example music. Slow, positive valence music has been shown to have a restorative effect on performance of the cognitively fatiguing Sustained Attention to Response Task (Baldwin and Lewis, 2017). The wave model interpretation is that slow music during a break period slows down attentional frequency and therefore provides restoration. Relatedly, Jones (1976, pp. 345–347) has discussed selective attentional emphasis of different levels of nested rhythms in music and speech. Attentional emphasis of slower nested rhythms is theorized to require less energy than faster rhythms. Also, slower rhythms are more likely to be emphasized because their higher level expectancies require more frequent adaptive adjustments to the temporally unfolding music or speech. As noted earlier, Large and Jones (1999) have modeled temporal expectancies as a tracking task in which an adaptively adjusted attentional oscillator anticipates the timing of an upcoming sequence of external events. This behavior corresponds to a higher level in Rosenberg's temporal hierarchy that is different from lower level spatio-temporal sampling of the environment. As noted by Busch and VanRullen (2010), environmental sampling does not require a rhythmic sequence of external events. When a rhythmic external sequence is present, both the lower level sampling and the upper level tracking will be active. Research on the coupling between these two adaptive oscillatory processes might provide a more detailed process model for the restoration effect of slow music. Additionally, such modeling might be helpful in research on rhythmic aspects of some clinical language disorders (e.g., Goswami, 2011) and language learning more generally (e.g., Campollo-Urkiza et al., 2026). Exposure to slow predictable rhythmic stimuli has been found to enhance language processing (e.g., Przybylski et al., 2013), and attention restoration may play some role in this effect. If so, other stimuli or behaviors known to restore attention may be helpful in enhancing language processing.

The present wave theory also predicts that attending a concert in a large arena or outdoor space may be particularly restorative due to the three properties that were ascribed above to natural forests—large spatial extent, fractal structure, and absence of distractions. Analysis of the frequency content of some musical compositions has found temporal self-similar fractal structure in their note sequences and/or audio power (e.g., Katcher and Wilkins, 1993; Charyton et al., 2012; Voss and Clarke, 1978). The wave theory predicts that both a large spatial setting and temporal fractal structure of some music would both induce listeners to lower their attentional frequencies and be sensitive to slower, restorative aspects of the music.

However, self-similarity by itself is probably not a sufficient condition for facilitating attention restoration. People express greater preferences for some visual fractal textures more than others (see Joye, 2007 for a review). Another example is provided by John Coltrane's jazz saxophone improvisations, which had fractal structure both early and later in his career as revealed in linear log-log plots of Fourier power vs. frequency in his note sequences (Charyton et al., 2012). However, the slopes of these linear relationships became flatter later in his career; his music became less predictable, less “connected,” and people found it less satisfying. Important questions for future research are what patterns of self-similarity enhance attention restoration, and how are these patterns related to the structure of human attention?

## 4 A wave interpretation of religious architecture and rituals

Kristof (2024), a New York Times columnist, extolls walking through the wilderness and recently offered the following comparison of natural settings and places of religious worship.

Some people worship in a church, others in a temple or mosque. I attend the cathedral of the wilderness, for among wildflowers in an alpine meadow we can all connect to something grander than ourselves…I see wild spaces as a place…to be dazzled and humbled by the vastness of space and the slowness of geologic time, …to bare our souls, to be recharged.

Kristof associates large distances with temporal slowing in the phrase “the vastness of space and the slowness of geologic time,” which is consistent with the present wave theory of distributed attention. The quotation suggests that there may be some similarities between experiences in nature and in religious settings, and the concept of attention waves might provide one way to explore such a comparison.

While Attention Restoration Theory has emphasized natural scenes, its possible application to particular built environments has also been a topic of interest, for example, see Joye (2007) for a review. The wave model of attention is abstract enough that it could apply to both natural and built environments, an example of which is religious architecture. In discussing monasteries, Kaplan (2001, p. 500) commented, “The architecture in such settings too appears to be designed with its restorative potential in mind.” Ouellette et al. (2005) and Herzog et al. (2010) performed factor analysis of questionnaire data to investigate people's motivations and subjective outcomes when respectively attending a monasterial retreat or houses of worship more generally. A number of the factors indicated that people find these experiences to be attentionally restorative. The wave model can provide some new hypotheses about how religious architectures and rituals contribute to these restorative effects.

Besides the above quote by Kristof (2024), a similarity between tall religious structures and forests is also captured in the description of a group of tall trees as a “forest cathedral,” for example, in Cook Forest, Pennsylvania, USA. The large dimensions of cathedrals, mosques, and temples combined with a lack of distractions can encourage long wavelength, low frequency attention waves, which can be restorative. Supporting experimental evidence comes from a study by Joye and Dewitte (2016) in which pictures of taller buildings induced greater feelings of awe and also slower responses in successively clicking on a set of small circular targets with a computer mouse. The authors interpreted this slowing as a “freezing” response. However, an alternative explanation is that taller buildings led to longer wavelengths with slower attentional frequencies, which led to slower targeted clicking and perhaps also facilitated slower higher level processes involved in feelings of awe.

A top-down, cognitively driven influence toward slow restorative attending can occur through religious rituals of prayer and chanting or through meditation more generally. Such practices often involve meditating on distant places or beings, for example, “our Father, Who art in heaven” in Christian prayer, praying toward the distant sacred site of Mecca in Muslim tradition, and imagining a rope that extends far upward from the top of one's head in a form of Kabbalah in Jewish tradition (Cooper, 2005). This common use of mental imagery that involves large distances could be a means of producing long wavelengths and slow attentional frequencies that are restorative. Additionally, DeSteno (2021) has reviewed how religious practices such as structured praying, reciting mantras, and singing hymns can result in physiological slowing of heart rate and breathing and mental calming. The slow rhythms of these practices may additionally lower attention wave frequency and therefore be restorative.

Religious architecture and rituals are designed to pull one away from many of the demands of everyday life and their attendant high frequency attention. In terms of Attention Restoration Theory, this aspect of religious practice would correspond to the concepts of Being Away and Extent, which involves “connectedness and scope” and can promote feelings of being “in a whole other world” (Kaplan and Kaplan, 1989, pp. 183–184). Overall, wave theory provides a coherent interpretation of how aspects of religious architecture and practice can contribute to restorative experiences in these built environments.

The search for a deeper understanding of attention can also benefit from inter-faith dialogue. For example, Zaleski (1994, 1995) reviewed attempts at Buddhist—Christian dialogue and suggested that more emphasis should be placed on comparing different practices for controlling attention rather than on differences in theological and cosmological beliefs. Zaleski noted that within both of these traditions attentiveness is important for achieving compassion and altruistic joy. Novak (2005, p. 605) has similarly written that “attention appears to be a *sine qua non* and common denominator of many of the forms of mental prayer and meditation…” The study of different religious practices may therefore contribute to research on attention if theories are sufficiently abstract to identify common underlying relationships.

## 5 Vigilance: attention restoration theory and human factors

The goals of vigilance research in the field of human factors have many parallels with attention restoration theory. Vigilance is defined as sustained attention in performing some task over a prolonged period of time (e.g., Warm et al., 2008). Early research on vigilance by Mackworth (1961) was aimed at understanding why the detection performance of radar and sonar operators searching for enemy craft in World War II could deteriorate over a half hour watch period. Such deterioration, referred to as “the vigilance decrement,” has subsequently been studied in many tasks including industrial inspection for flaws, air traffic control, driving, monitoring patient status during surgery, and baggage inspection at airports (see Warm et al., 2008 for a review). Breaks from the primary task are known to alleviate the vigilance decrement (Mackworth, 1961). An interesting issue is how to structure the breaks to restore performance as much as possible (Helton and Russell, 2015).

Attention Restoration research has also studied vigilance tasks. For example, a recent meta-analysis by Stevenson et al. (2018) reviewed five vigilance experiments and did not find evidence for a significantly larger restoration effect for exposure to images of nature vs. control conditions. The cognitively demanding tasks were the Sustained Attention to Response Task (quickly responding to all but one particular numerical digit), the Multi-Source Interference Task (quickly reporting which of three numbers is different from the other two identical numbers), and the Attention Network Task (quickly reporting the direction of an arrow with variations in temporal and spatial pre-cues and distracting stimuli). Other meta-analyses by Ohly et al. (2016) and Bell et al. (2025) also did not find evidence for exposure to nature improving vigilance performance relative to control conditions. Stevenson et al. (2018) also compared the effectiveness of exposure to images of nature vs. exposure to actual natural environments in restoring performance on cognitively fatiguing tasks that strongly involved attention control, working memory, or cognitive flexibility. For these types of tasks, images of nature did not significantly contribute to a restoration advantage over control conditions on average across experiments, whereas actual exposure to natural environments did significantly contribute to an enhanced restorative effect.

The above results regarding vigilance tasks and exposure to nature are disappointing given the importance of the vigilance decrement in numerous real world tasks. One suggestion for additional research on exposure to nature or other interventions is to replace brief artificial laboratory tasks for inducing fatigue with tasks that more closely resemble real world vigilance tasks. In making this point, Mackie (1987) reviewed research in which critical findings regarding a vigilance decrement did not become evident until after 2–4 h of a sonar style signal detection task and after several successive days of a 7–9 h driving task. The effectiveness of simple rest breaks for restoration from brief laboratory vigilance tasks can be so strong that there is a ceiling effect that reduces sensitivity for measuring any additional contribution from exposure to nature (e.g., Finkbeiner et al., 2016). Simple rest breaks can be less effective for very extended vigilance tasks such as pilots performing long distance flights (Neri et al., 2002), which provide a better chance to detect an added contribution from exposure to nature.

Additionally, the theoretical perspective provided by the wave model of attention may be helpful in facilitating greater cooperation across the fields of human factors and attention restoration. Viewing images of natural scenes can restore attention (Berman et al., 2008; Jiang et al., 2023), but such effects tend to be smaller and less reliable compared to actually being in a natural environment (e.g., Jaffe, 2010; Stevenson et al., 2018). With the aid of augmented or virtual reality technology (Anderson et al., 2017), a person could wear a head mounted display, and the apparent size and depth of images of natural scenes could be increased far beyond the limitations of computer screens that are typically used for such research. Movement in such virtual environments can also be simulated with a treadmill (Crossan and Salmoni, 2021). The wave model predicts that larger images of nature should be more effective at increasing attentional wavelength and thereby slowing attentional frequency and increasing attentional restoration. Smaller images would be expected to encourage high frequency attention, and thereby work against the potential benefit of fractal images associated with nature. The present model does not predict how long a break needs to be to restore attention, which is likely to be task specific. If successful, the use of large virtual reality depictions of nature might be useful in industrial, aviation, medical, and other applied vigilance contexts (Warm et al., 2008). Attention Restoration Theory could enhance human factors research, and in turn Attention Restoration Theory could benefit from greater statistical power to detect nature's influence.

Additionally combinations of top-down techniques such as meditation and bottom-up techniques such as expansive natural scenes involving multiple sensory modalities could be investigated to see if their combined effects are larger than their individual effects in strengthening attention restoration (e.g., Kaplan, 2001). If different sensory modalities each have distinct attentional resources (Wickens, 2008), their wave-like structure for distributed attention may provide a mechanism for temporally aligning environmental samples from these multiple sources to form a coherent perceptual whole. This temporal alignment would synchronize the ensemble of traveling waves and permit their frequencies to be lowered together in response to stimulation from various modalities. Non-linear dynamical modeling of this synchronization process, for example, Jackson (1991), might offer insight into how some combinations of attention restoration techniques may be more stable.

## 6 High frequency attention to modern technology and distracting events

In terms of the wave model, sudden forceful environmental events can easily disrupt low frequency attention and replace it with focused or high frequency distributed attention to examine the source of the environmental stimulus. The stability of low frequency, long wavelength attention can be fragile. Loud conversational talking in a temple, mosque, or cathedral is disruptive of long wavelength attention associated with prayer or meditation. Similarly, loud conversational talking is disruptive of the silence of the wilderness that allows hikers to engage in low frequency, long wavelength attention. The loud noises associated with motorboats, motorcycles, and helicopters can be similarly disruptive in places where people seek silence.

In contrast to the restorative style of attending, everyday use of cell phones and computers typically involves attending to small images at high event rates as people rapidly scroll from one topic to another. Rapidly changing cell phone and computer displays can be mentally exhausting. Additionally, their use in a natural setting can substantially diminish the restorative attentional effect that could have been gained (Jiang et al., 2019). Carr (2010) suggests that this style of attending to modern technology tends to dominate modern life and makes it difficult for people to attend deeply to a single topic for prolonged periods. The generally fast pace of modern life may also diminish opportunities for sustained attention. As noted by Novak (2005, p. 606) in his review of religious meditative traditions,

Of primary importance is the discovery that contemplative practices universally require the aspirant to develop an attentional capacity that, unlike his or her ordinary attention, is relatively non-discursive, uncapitulatingly alert, and sustained.

Increasing the stability of low frequency attention from various distractions may require training and practice as exemplified in various religious practices.

A general concern with modern technology is that although high frequency distributed attention and focal attention are inherently useful and important, they may dominate people's daily existence to an extent that there is very little low frequency distributed attention. Lack of low frequency distributed attention may leave people attentionally fatigued and thereby diminish the quality of their cognition and their overall quality of life. Perhaps a better understanding of attentional dynamics in the context of Attention Restoration Theory can improve our evaluation of how we use technology and help people find a better balance between restorative low frequency attention and fatiguing forms of attention.

## 7 Summary

Research indicates that sustained focal attention temporally samples the environment. For distributed attention, the additional need to sample the environment both temporally and spatially can be accomplished with traveling waves, examples of which have been found in many parts of the brains of different species. It is proposed that the spatial and temporal frequencies of attention waves can be lowered in environments with large distances, slow temporal rhythms, and fractal structure, which can restore attention from fatigue. This conceptualization provides a theoretical model for interpreting the relevance of Attention Restoration Theory to outdoor experiences in natural settings, various forms of meditation, aspects of music, the structure of some built environments, attentional aspects of religious practice, vigilance tasks, and the evaluation of modern technology. It is hoped that this wave theory of distributed attention will promote more quantitative, interdisciplinary research on Attention Restoration Theory and expand both its domain and the domain of cognitive and environmental psychology more generally.

## Data Availability

The original contributions presented in the study are included in the article. Further inquiries can be directed to the corresponding author.
